# Facile fabrication of microparticles with pH-responsive macropores for small intestine targeted drug formulation

**DOI:** 10.1016/j.ejpb.2018.05.014

**Published:** 2018-07

**Authors:** Bahman Homayun, Chengmeng Sun, Ankit Kumar, Carlo Montemagno, Hyo-Jick Choi

**Affiliations:** a Department of Chemical and Materials Engineering, University of Alberta, Edmonton, AB T6G 1H9, Canada; b Southern Illinois University, 1263 Lincoln Dr, Carbondale, IL 62901, USA

**Keywords:** Oral administration, Drug delivery, Microparticles, Macropore, Gastrointestinal tract, pH, Emulsion

## Abstract

Oral drugs present the most convenient, economical, and painless route for self-administration. Despite commercialization of multiple technologies relying on micro- and nanocrystalline drugs, research on microparticles (MPs) based oral biopharmaceuticals delivery systems has still not culminated well enough in commercial products. This is largely due to the drugs being exposed to the destabilizing environment during MP synthesis process, and partly because of complicated process conditions. Hence, we developed a solvent swelling-evaporation method of producing pH-responsive MPs with micron-sized macropores using poly(methacrylic acid-co-ethyl acrylate) in 1:1 ratio (commercial name: Eudragit® L100-55 polymer). We investigated the effects of temperature and evaporation time on pore formation, freeze-drying induced pore closure, and the release profile of model drugs (fluorescent beads, lactase, and pravastatin sodium) encapsulated MPs in simulated gastrointestinal tract conditions. Encapsulated lactase/pravastatin maintained >60% of their activity due to the preservation of pore closure, which proved the potential of this proof-of-concept microencapsulation system. Importantly, the presence of macropores on MPs can be beneficial for easy drug loading, and solve the problem of bioactivity loss during the conventional MP fabrication-drug encapsulation steps. Therefore, pH-sensing MPs with macropores can contribute to the development of oral drug formulations for a wide variety of drugs and bio-macromolecules, having a various size ranging from genes to micron-sized ingredients with high therapeutic efficacy.

## Graphical abstract

**Figure:**
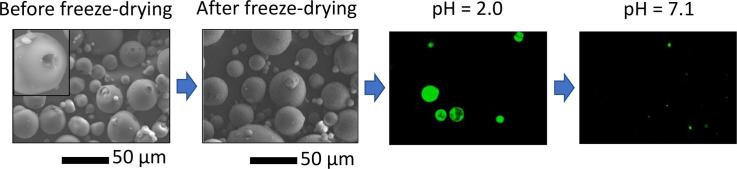

## 1 Introduction

Microparticles (MPs) are widely utilized as delivery vehicles in food, pharmaceuticals, and cosmetics industries due to their capability of encapsulating substances, preserving them in unfavorable conditions, and delivering them at the desired target sites [1], [2], [3], [4], [5]. These carriers are broadly classified into two main categories: solid MPs that have drug molecules dispersed in their polymer matrix and hollow MPs with a hollow interior to encapsulate the pharmaceutical ingredients [6], [7]. Application-specific functionalization of microencapsulation systems allowed for the production of smart delivery carriers, which respond to the surrounding stimuli (e.g., temperature and pH) [6], [8], [9], [10], [11]. For oral drug delivery applications, pH-responding carriers are ideal candidates. This is due to their pH-dependent ionization/deionization behavior, which can be utilized for the protection of the drugs/biopharmaceuticals in the unfavorable pH conditions and subsequent delivery to the target sites [12], [13].

A number of MP fabrication methods, such as phase separation, spray-drying, and emulsion-solvent evaporation/extraction have been employed to realize the desired features and characteristics in drug delivery carriers [14], [15], [16], [17], [18]. The key idea of phase separation method is adding a solvent, which cannot dissolve the drug or the polymer, but is miscible with the solvent containing the drug and polymer. Adding the non-solvent causes the phase separation of the polymer, which leads to the formation of coacervate droplets [19]. However, the use of organic solvent will inevitably raise concerns about both the safety and destabilization of therapeutic biomolecules [20], [21]. In the case of the spray-drying method, the fast evaporation of the organic solvent in the drying chamber can be advantageous for the formation of fine MPs with reduced exposure of drugs to the organic solvent [22]. However, this method may involve complicated optimization of the fabrication parameters such as feed rate, temperature, and concentration of different types of excipients and drugs [22], [23], [24]. Emulsion-solvent evaporation/extraction method has been most widely employed to fabricate MPs. In this method, the organic solvent can be removed from the emulsion by either leaching the volatile organic solvent in the dispersed phase (solvent evaporation) or by transferring the emulsion to a quenching medium (solvent extraction) for the solidification of MPs [25], [26], [27]. However, the main drawbacks of these systems are associated with the low throughput, insufficient encapsulation ability, inadequate protection of the drugs in the harsh environment, and inefficient release profiles at the target sites [28], [29], [30], [31], [32]. Particularly, the main problem is related to the efficacy of encapsulated drugs. During the encapsulation process, biopharmaceuticals are inherently exposed to the destabilizing elements, such as organic solvents, O-W interface, and hydrophobic interaction with the polymer matrix, because of the process limitations [33], [34], [35], [36], [37], [38].

To resolve these problems, Kumar et al. in our group developed MPs with a pH-responsive macropore using a new O/W emulsion/solvent extraction protocol. In pored MPs made of an anionic copolymer (Eudragit® S100, abbreviated as S100), the presence of surface pores is responsible for the direct and solvent-free encapsulation, thus avoiding destabilization of the drugs during the loading process [29]. MPs with macropore exhibited a wide range of advantages originating from their unique architecture, such as the easy loading of drugs and pH-modulated pore opening-closing behavior. Although the few micron-sized MPs developed by Kumar et al. had the capability for the encapsulation of large-size ingredients (i.e. 100 nm drug), bigger MPs with micron-scale pores promise for the encapsulation and delivery of even larger-sized biopharmaceuticals, such as viruses and bacteria and offer an excellent platform for the administration of large doses of drugs. Nevertheless, increasing the size of the droplets in the emulsion drastically decreases the Laplace pressure inside the particles prepared through the emulsion solvent evaporation/extraction techniques, leading to the destabilization of the emulsion droplets [39]. Therefore, the development of a new process to fabricate large MPs with pH-responsive macropores is recognized as another critical challenge for the successful implementation of the oral drug delivery systems.

This research aims to develop an innovative solvent evaporation method for fabricating MPs with pH-responsive macropores that can be used for the small intestine-targeted delivery of oral drugs to the small intestine. Here, we report a facile technique to manufacture MPs with macropores by utilizing the Eudragit® L100-55 polymer (hereafter referred to as L100). This is based on our observation that the L100 polymer powder consists of MPs with a few micron-sized surface pores. The size of these MPs ranges from a few to tens of micrometers. Hence, our approach was to devise a facile method that can modify the original powder to have macroscopic pores, and use them as an oral drug delivery system. As a biocompatible FDA approved enteric formulation, L100 is an anionic environmental-sensitive copolymer with pKa around 5.5, which is not metabolized in the body, remains stable at the gastric environment, and responds to intestinal pH. The original polymer powder, which consists of anionic MPs, was incubated in a single organic phase, dichloromethane (DCM). The reasons for selecting DCM include: 1) the insolubility of the polymer in DCM, 2) the small size of the solvent molecule, which facilitates its diffusion through the polymer matrix, and 3) its low boiling point (39.6 °C), which is much lower than the glass transition temperature (T_g_) of the polymer. The suspension went through the solvent evaporation step to form and enlarge the interior void space and the surface pores present in the original polymer particles. This approach incorporates the critical parameters previously investigated in the solvent evaporation method for the creation of surface pores in the MPs [29], [40]. As such, the effects of solvent evaporation temperature and the incubation time were evaluated to test the pore size controllability in the MP system. The encapsulation capability of the MPs with macropores was tested by using three different sizes of fluorescent particles (i.e. 100 nm, 1 µm, and 4 µm), and their pH-dependent release behavior was monitored in the simulated GI conditions. The protective efficacy of the MPs was assessed by measuring the remaining activity of β-galactosidase, an enzyme used as a supplement for the lactose intolerance, using the *ortho*-nitrophenyl-β-galactoside (ONPG) assay. Lastly, the viability of the pored MPs as a delivery system was further confirmed by measuring the pH-dependent release profiles through pore closure/opening and the capability of preserving the structural stability of an encapsulated drug, pravastatin sodium [HMG-CoA (3-hydroxy-3-methylglutaryl-coenzyme A) reductase inhibitor]. For this purpose, the high-performance liquid chromatography-mass spectroscopy (HPLC-MS) and HPLC- UV/Vis methods were utilized.

## 2 Methods

### 2.1 Materials

Poly(methacrylic acid-co-ethyl acrylate) in 1:1 ratio, commercially known as Eudragit® L100-55 (hereafter referred to as L100), was obtained from Evonik Canada Inc. (Burlington, Ontario, Canada). 2-Nitrophenyl β-D-galactopyranoside, β-galactosidase from Aspergillus Oryzae, galactose and lactose assay kit, sodium dodecyl sulfate, acetonitrile, and disodium hydrogen phosphate were acquired from Sigma-Aldrich (St Louis, Missouri, USA). Yellow-green fluorescent beads with different sizes (100 nm, 1 µm, and 4 µm) were purchased from Life Technologies (Carlsbad, CA, USA).

### 2.2 Fabrication of pored MPs and pore closure by freeze-drying

To test the effects of temperature on MPs, 5 g of L100 polymer (abbreviated as MP_Original_) was added to 100 mL DCM in a beaker and then vortexed for 4–5 s. The aliquots of the polymer-DCM suspension were stir-dried overnight at the room temperature (RT). Also, aliquots of the polymer-DCM suspension (10 mL) were put into 250 mL Petri-dishes for solvent removal by incubation in an oven under two different conditions: (1) incubation at 65 °C for 30 min, followed by overnight incubation at 37 °C, and (2) overnight incubation at 37 °C.

To investigate the effect of incubation time of L100 polymer-DCM mixture on the morphology of MPs, the suspension in the sealed container was further stir-incubated at 39 °C (50–60 rpm) in a water bath. This is based on the prediction that the temperature just below the boiling point of the DCM would maximize the diffusion of the organic phase into the polymer matrix. Aliquots (10 mL) were taken over the course of several incubation time intervals (0, 30, and 120 min; abbreviated as MP_0 min_, MP_30 min_, and MP_120 min_, respectively). These samples were dried in a glass petri-dish in an incubator (Isotemp Incubator, Thermo Fisher Scientific). After the initial drying at 65 °C for 30 min, the temperature was brought down to 37 °C for overnight incubation. Subsequently, MPs were collected for further analysis.

Pores on MPs were closed by utilizing a freeze-drying method, following the protocol developed by Kumar et al [29]. Briefly, 20 mg of each sample was suspended in 1 mL DI water, which was then frozen in the liquid nitrogen and freeze-dried using the protocol reported in the above-mentioned study. (AdVantage Pro Freeze Dryer, SP Scientific; Warminster, PA). The presence of residual DCM of MPs was investigated using Fourier transform infrared (FTIR) spectroscopy (Thermo Nicolet NEXUS 870 FTIR ESP, Thermo Fisher Scientific).

### 2.3 Preparation and pH-dependent release behavior of fluorescent bead-encapsulated MPs

Encapsulation ability of MPs based on the size of the ingredients was tested using three different types of fluorescent beads: 100 nm, 1 µm, and 4 µm. In a typical procedure, 50 mg of MP sample from each condition, i.e. MP_Original_, MP_0 min_, MP_30 min_, and MP_120 min_, was added to 1 mL carboxylate-modified yellow-green fluorescent bead solution. The bead solution had been 10-fold diluted in DI water from the original product. Vacuum on/off cycle was applied four to five times to lower the surrounding pressure, replace the air pockets inside the MPs with the ingredients solution and fluorescent beads through the surface pore [29]. Fluorescent bead-encapsulated MPs were subsequently centrifuged at 500 RCF for 2 min (Eppendorf Model 5810; Hamburg, Germany) and collected. Then, the pellets were resuspended in DI water, rapidly frozen in liquid nitrogen, and freeze-dried. Fluorescence microscopy was employed to observe and analyze fluorescent bead-encapsulated MPs.

Freeze-dried samples were washed with a potassium chloride (KCl, 0.2 M)/hydrochloric acid (HCl, 0.2 M) buffer at pH 2.0 (simulated gastric fluid, abbreviate as SGF) twice. Washed MPs were suspended in the same buffer at 37 °C for 2 h to simulate the digestion process of the stomach. The samples were subsequently incubated in a solution composed of KCl (0.05 M), HCl (0.05 M), and Na_2_HPO_4_ (0.1 wt%, pH 7.1) at 37 °C for 4 h (simulated intestine fluid, abbreviated as SIF). Throughout the whole simulated GI tract digestion process, fluorescence emission was measured every 10 min to monitor the time-dependent release behavior of the fluorescent bead-encapsulated MPs. The excitation wavelength was kept at 490 nm and the emission wavelength was between 500 and 530 nm, with a step size of 1 nm.

### 2.4 Preparation and release/stability tests of lactase-encapsulated MPs

For the encapsulation and stability tests using lactase enzyme, MPs prepared after the incubation for 120 min (MP_120 min_) were used. This was due to their highest pore/particles size ratio as well as the highest loading ability for larger ingredients. Similar to the encapsulation process for the fluorescent beads, 1 g of MP_120 min_ sample was suspended in the lactase enzyme-containing formulation (20 mg/mL lactase enzyme, 15 wt% trehalose, 5 wt% carboxymethyl cellulose), followed by vacuum cycles for the encapsulation and freeze-drying for the pore closure [29].

After the washing step in SGF solution, the lactase-encapsulated sample was incubated in the simulated GI tract conditions, as described in Section 2.3. The concentration of the released enzyme was measured using micro BCA assay kit, and the activity of the released enzyme was assayed using a 2-nitrophenyl β-D-galactopyranoside (ONPG), following the previously reported protocol [29].

### 2.5 Pravastatin sodium encapsulation and release/stability tests

HPLC-UV was performed to determine the stability of pravastatin sodium (hereinafter abbreviated as pravastatin) in the test conditions over time, using pravastatin suspended in both DI water and pH 7.1 solution.

For the acquisition of pravastatin calibration curve, a set of 10–50 μg/mL pravastatin in pH 7.1 solution was prepared with a step size of 10 μg/mL, and another set of 2–10 μg/mL pravastatin in pH 7.1 solution with a step size of 2 μg/mL. To subtract other chemical signals in the matrix, HPLC-UV from pH 7.1 solution was also obtained as a background signal. Solution preparation and HPLC-UV test were performed in triplicate for all concentrations to construct pravastatin calibration curve.

Pravastatin powder (25 mg) was dissolved in 5 mL of DI water. After the preparation of the pravastatin stock solution, 40 mg of MP_120 min_ was suspended with 1 mL of pravastatin stock solution contained in a 2 mL Eppendorf tube, followed by the same vacuum cycles and freeze-drying conditions described earlier in 2.2, 2.3. After freeze-drying, the samples were collected and stored at 4 °C in a refrigerator for further release tests.

Samples were prewashed before each release test. Pravastatin-encapsulated MP pellet was suspended in 4 mL of acidic buffer (pH 2.0) with gentle shaking in a water bath at 37 °C for 2 h. 250 μL was aliquoted every 30 min for centrifugation at 5,000 RCF for 2 min. Then the supernatant was transferred for filtration through 0.2 µm pore-size regenerated cellulose filters (Corning, Inc., Germany). The filtrate (100 μL) was mixed with 350 μL of 0.1 wt% disodium phosphate (Na_2_HPO_4_) solution in a new 1.5 mL tube for HPLC-UV and HPLC-MS tests. At the beginning of incubation in the acidic buffer, another 250 μL of the sample taken from 0 min sample is mixed with 875 μL of disodium phosphate solution, incubated for the whole 6-h incubation process as an indication of the maximum encapsulated pravastatin. After 2 hr of incubation in SGF, pH of the sample was controlled to 7.1 for additional 4-h incubation in SIF. Every 30 min, 1 mL of the sample was aliquoted for HPLC-UV and HPLC-MS analysis. 40 mg of MP_120 min_ without pravastatin was used as the background.

### 2.6 Characterization methods

#### 2.6.1 Scanning electron microscopy (SEM)

The morphology of the MPs was characterized using the scanning electron microscope (SEM; S4800 Electron Microscope; Hitachi, Japan). The pore-particle size ratio of the MPs (acquired from 300 measurements) was measured from the image analysis using Adobe Photoshop CS3. The image analysis was performed to study the effects of the fabrication protocol on the samples, the effects of solvent evaporation on the pore formation or pore extension in MPs, and to evaluate the pore closure efficiency of the freeze-drying process. The SEM images were also used for measuring the number ratio of the number of pored particles divided by the total number of particles to monitor the effect of the solvent evaporation process on the extension or formation of the surface pores on MPs. In a typical procedure, MPs in the powder form were placed uniformly on a double-sided carbon tape. The sample was coated with a 7-nm gold layer to minimize the charging effect and observed at 15 kV (20 µA). To observe the pH-dependent change in morphology of MPs, 40 µL of the sample solution obtained at each time interval. This solution was carefully dropped on a glass coverslip and the major portion of the water was quickly removed by a filter paper using the blotting technique, and the sample was further dried in a fume hood at RT.

#### 2.6.2 Fluorescence microscope

An Olympus IX81 inverted microscope (Olympus, Germany), coupled with a DP 80 digital camera and dual CCD sensor, was used for the fluorescence microscopy analysis. The images were recorded at the 40× objective (Olympus LCPlanFl, 1 µm depth of field, NA 0.6) using CellSens software (Olympus, Germany). Sample solutions obtained from the release test at different time intervals were placed on a glass slide, covered with a glass coverslip, and imaged using FITC filter sets.

#### 2.6.3 High-performance liquid Chromatography-Mass spectroscopy (HPLC-MS)

To confirm the presence of pravastatin and its 3′α-isoform, Agilent 1100 series with LC/MSD detection was employed [41]. The experiment was performed in the positive mode with a scan range of 100–800 m/q. HPLC separation of the drugs was achieved via a Zorbax SB-C18 column (5 μm, 4.6 × 250 mm) (Agilent Technologies, Inc.; Santa Clara, CA, USA) using a mobile phase composed of 0.1 wt% formic acid in a mixture of DI water and acetone nitrile (3:1 v/v) at a flow rate of 0.5 mL/min (samples injection volume: 20 μL). HPLC-MS tests were performed on pravastatin-encapsulated in MP_120 min_, which were incubated for 6 h in the simulated GI tract pH conditions. Pravastatin (50 μg/mL) in pH 7.1 buffer and MPs in pH 7.1 buffer were used as a control and a sample blank, respectively.

#### 2.6.4 High-performance liquid Chromatography—UV/Vis (HPLC-UV/Vis)

HPLC-UV/Vis was performed using Agilent 1100 series with the same column as HPLC-MS test. Na_2_HPO_4_ (25 mM) with 1 mM of sodium dodecyl sulfate was dissolved in DI water, and then mixed with acetonitrile (aqueous: acetonitrile (v/v) = 3:1) as an HPLC mobile phase. The detection wavelength was set at 238 nm with a reference of 100 and 600 nm (flow rate: 0.5 mL/min, injection volume: 20 μL). Testing of the released samples at each time point was performed in triplicate.

### 2.7 Statistics

Student’s *t*-test and One-way ANOVA in Minitab software (State College, PA, USA) were utilized for analyzing the data. A *P*-value of less than 0.05 implied a significant difference.

## 3 Results

Previous work reported a technique to produce MPs with a pH-responsive single pore on their surfaces by controlling the evaporation rate of the organic solvent in the emulsion [29]. The protocol was effective in fabricating hundreds of nanometer-sized macropore on MPs of a few micrometers in size. However, it was not successful in producing MPs with micron-sized macropores. In this study, swelling of original L100 particles with dichloromethane (T_b_: 39.6 °C, abbreviated as DCM), followed by the solvent evaporation was used to form new pores and/or increase the size of the existing pores. The effects of process parameters on forming MPs with micron-sized macropores and their potential as a small intestine-targeted drug delivery system has been evaluated below.

### 3.1 Fabrication of MPs with macropores

#### 3.1.1 Effect of evaporation temperature on MPs and pore closure process

**Fig. 1:**
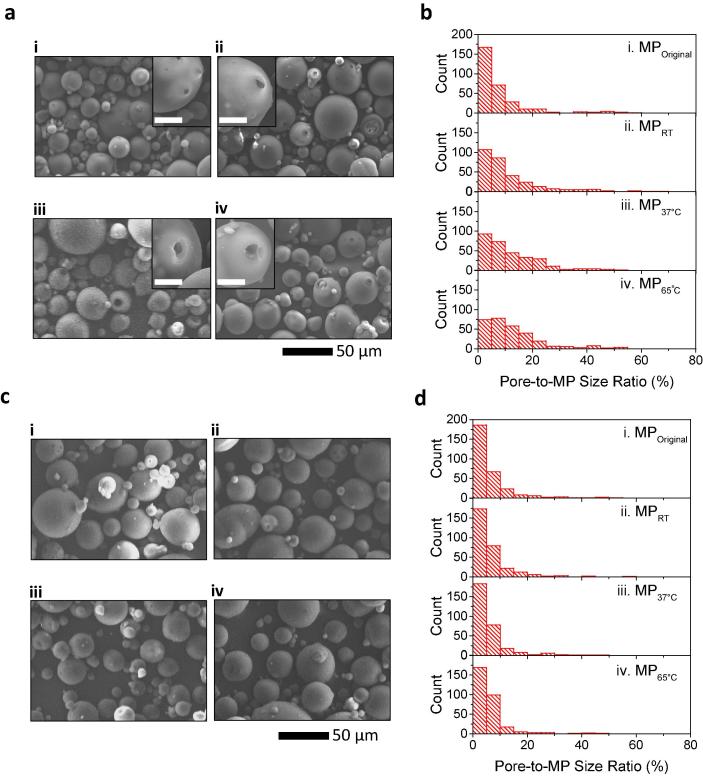
Effect of evaporation temperature on the fabrication and size distribution of MPs with macropores. (a) Histogram of pore-to-MP size ratio and (b) its corresponding SEM images: (i) MP_Original_, (ii) MP_RT_, (iii) MP_37°C_, and (iv) MP_65°C_. Effect of freeze-drying on pore closure. (c) Histogram of pore-to-MP size ratio measured from MPs with unsealed pores and (d) its corresponding SEM images: (i) MP_Original_, (ii) MP_RT_, (iii) MP_37°C_, and (iv) MP_65°C_. Size distributions in (a) and (c) were analyzed from SEM images (*n* = 300).

To determine the effects of temperature on the pore formation and its size change, L100 particles (original MPs with the average diameter of 26.5 ± 12.4 µm, MP_Original_ with pores) in DCM suspension was evaporated at three different temperatures (i.e. overnight incubation at RT, MP_RT_; overnight incubation at 37 °C, MP_37°C_; and incubation at 65 °C for 30 min followed by overnight incubation at 37 °C, MP_65°C_). The morphological change of both MPs and pores were characterized by SEM. Fig. 1a shows selected SEM images of (i) MP_Original_, (ii) MP_RT_, (iii) MP_37°C_, and (iv) MP_65°C_. It is evident that the solvent evaporation has a dominant effect on the pore formation compared to the control (i.e. MP_Original_). As shown in histograms for pore-to-MP size ratio in Fig. 1b, compared to MP_Original_ (i), all solvent evaporation conditions (i.e. ii, iii, and iv) induced the increase in the population of MPs with large pores (One-way ANOVA, *P* < 0.001), indicating an important role of solvent evaporation process in controlling the size of pores in MPs. In addition, the pore-to-MP size ratio histogram shifted toward the large size, implying an increase in the size of pores with evaporation at a higher temperature, which is consistent with SEM images in Fig. 1a. This suggests a strong correlation between solvent evaporation temperature and pore size (the average diameters of the pores: 2.1 ± 2.5 µm (MP_Original_), 2.9 ± 3.0 µm (MP_RT_), 3.1 ± 2.6 µm (MP_37°C_), and 3.4 ± 2.8 µm (MP_65°C_)). A similar trend was observed for the pored particle-total particle number ratio (see Fig. S1). That is, the distribution peak shifted toward higher values with the increase of incubation temperature (One-way ANOVA, *P* < 0.001). This means that solvent evaporation process may contribute not only to the increase in the size of pores but to the formation of new pores in the MPs. Moreover, the effect was observed predominantly from MP_65°C_ conditions. Thus, a triggered efflux of solvent molecules out of the L100 particles is thought to have led to enhanced pore formation and pore expansion due to higher evaporation rate (i.e. higher evaporation temperature).

Considering the application of MPs with macropores in pH-sensitive pharmaceuticals, pore closing behavior of the MPs upon freeze-drying was investigated by measuring the change in pore size and the population of pored particles from SEM analysis (see Fig. 1 c and d for microscope images and pore-to-MP size distribution, respectively). As shown in Fig. 1c, it is evident that pore closure occurred in a majority of MPs, indicating that freeze-drying process effectively closed the pores of MPs. This is supported by the significant decrease in the population of pored MPs after freeze-drying, as analyzed from SEM image analysis (compare Fig. S1 (before freeze-drying) with Fig. S2 (after freeze-drying)). Moreover, partially sealed pores showed a decrease in their size due to freeze-drying (One-way ANOVA, *P* < 0.001), as evidenced by the increase in the population of MPs with smaller pore sizes (i.e. left shift of the histograms of unsealed pore-to-MP size ratio) compared to those prior to freeze-drying (compare Fig. 1d with Fig. 1b). These findings are consistent with our previous report on pore sealing by freeze-drying [29], and further supports the applicability of this method in sealing the pores of L100 MPs with micron-sized pores.

For the application of pored MPs in the delivery of diverse biopharmaceuticals, it is critical to create large pores for easy drug loading and maintain high pore closure efficiency for drug protection at the acidic pH. Based on the pore-MP size ratio and pored particle-total particle number ratio analyses, it is reasonable to assume that MPs with macropores prepared at the 65 °C (30 min)/37 °C (overnight) conditions, i.e. MP_65°C_, represent the most promising among those tested for the general application.

#### 3.1.2 Effects of preincubation in solvent on pore formation

**Fig. 2:**
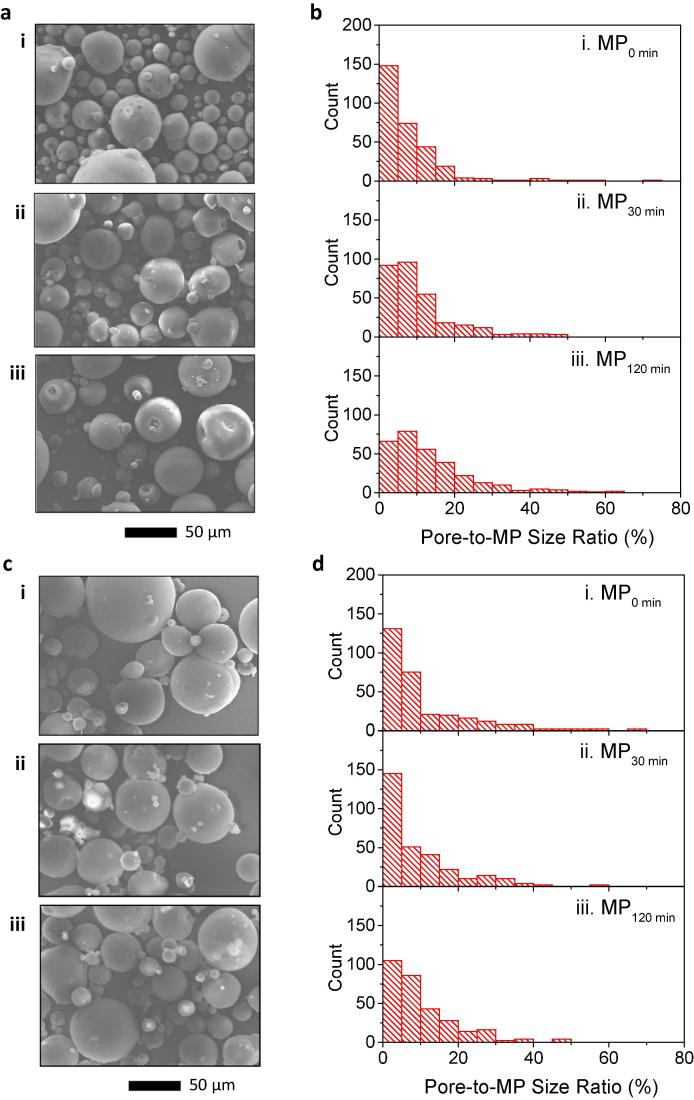
Effects of preincubation in DCM on pore formation. (a) Histogram of pore-to-MP size ratio and (b) its corresponding SEM images: (i) MP_0 min_, (ii) MP_30 min_, and (iii) MP_120 min_ (*n* = 300). (c) Histogram of pore-to-MP size ratio measured from MPs with unsealed pores after freeze-drying and (d) its corresponding SEM images: (i) MP_0 min_, (ii) MP_30 min_, and (iii) MP_120 min_ (*n* = 164).

To further evaluate the effects on the pore size by swelling in DCM solvent, L100 powders were preincubated at 39 °C for three different time intervals (0 min, 30 min, and 120 min) prior to solvent removal by incubation at 65 °C (30 min)/37 °C (overnight). Their pore-to-MP size ratio was characterized by SEM analysis. The as-prepared samples were abbreviated as MP_0 min_, MP_30 min_, and MP_120 min_, respectively. As observed in Fig. 2a, pores in MPs were greatly expanded with the longer incubation time in the solvent. As shown in the histograms for the pore-to-MP size of Fig. 2b, the population peak shifted to the right with the increase of preincubation time, indicating the increase of pore size with incubation (One-way ANOVA, *P* < 0.001). The average diameters of the pores were measured to be 1.8 ± 1.6 µm (MP_0 min_), 2.2 ± 1.6 µm (MP_30 min_), and 3.1 ± 2.8 µm (MP_120 min_) from samples at three different incubation time in DCM before solvent evaporation (see Fig. S3). As a result, 120 min incubation in DCM, followed by evaporation at 65 °C for 30 min and at 37 °C overnight, generated the most pored MPs with the largest pore size among all the conditions tested in this study. These results suggest that the size of MP surface pores strongly depends on the incubation time in the solvent. Furthermore, a higher degree of swelling of L100 polymer in DCM, followed by evaporation, plays an important role in producing MPs with larger pores. This can be further supported by comparing SEM images of fractured MP_Original_ and MP_120min_ samples (see Fig. S4 for representative SEM images).

Freeze-drying of the MP samples resulted in two types of MPs: MPs with closed pores and few MPs with unsealed pores (see Fig. 2c for SEM images of freeze-dried MPs and 3d for the size distribution of remaining pores). Similar to the observation in Fig. 1c and d, while the majority of freeze-dried MPs exhibited closure of their pore-mouth, MPs with incomplete pore closure also showed a significant decrease in their pore size due to freeze-drying (compare Fig. 2c and a for morphological evolution of pored MPs, and Fig. 2b and d for pore-to-MP size distribution). As shown in Fig. S3, the average diameter of unsealed pores was analyzed to be 1.5 ± 1.2 µm (MP_0 min_), 1.8 ± 1.2 µm (MP_30 min_), and 2.0 ± 1.5 µm (MP_120 min_). These analyses clearly demonstrate the effectiveness of the freeze-drying process in sealing the pores of MPs.

### 3.2 Encapsulation and release test of MPs with macropores

#### 3.2.1 Fluorescent beads-encapsulated MPs

Three different sizes of fluorescent beads (i.e. 100 nm, 1 µm, and 4 µm) have been incorporated into MPs through their surface pores by applying vacuum/release cycles, in order to evaluate their encapsulation/release characteristics. Subsequently, MPs were exposed to the simulated GI tract pH conditions for monitoring time-dependent release behavior.

**Fig. 3:**
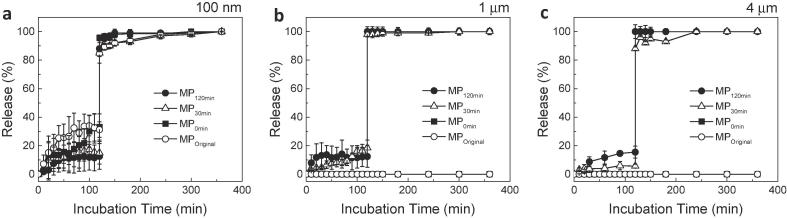
Release of fluorescent beads from MPs in simulated GI conditions. Time-dependent release profile of encapsulated (a) 100 nm fluorescent nanobeads, (b) 1 µm microbeads, and (c) 4 µm microbeads from MPs (MP_Original_, MP_0 min_, MP_30 min_, and MP_120 min_) subjected to simulated GI tract environment (SGF: 2-h incubation at pH 2.0 and 37 °C, and SIF: 4-h incubation at pH 7.1 and 37 °C). Release rate of fluorescent beads was calculated by measuring the relative fluorescent intensity of a sample (i.e. fluorescent intensity of a sample relative to that of a control with a complete release at pH 7.1). To achieve a complete release, fluorescent bead-encapsulated MPs were vortexed for 5 min after 4-h incubation at pH 7.1. (*n* = 5, mean ± SD).

Fig. 3 represents release profiles measured from fluorescent beads-encapsulated MPs by monitoring fluorescence intensity changes (Fig. 3a: 100 nm, Fig. 3b: 1 µm, and Fig. 3c: 4 µm). As shown in Fig. 3a, all MP samples could successfully encapsulate 100 nm-sized fluorescent nanobeads and remain intact in SGF. On the other hand, micron-sized beads could be only encapsulated into MP_30 min_ and MP_120 min_, which can be explained by the presence of larger pores (>2 µm) as shown in Fig. 2a and b (see Fig. 3b and c for time-dependent release profile).

The examination of the release profile also shows that within 20 min, MPs exhibit initial release of fluorescent beads upon exposure to SGF, followed by a slow rise or saturation in the release profile of up to 5–34% over the course of 2 h-incubation. Transferring the bead-loaded MPs to SIF, however, led to a faster release of beads (∼90–100%) within 10 min. The initial release profile during 2-h incubation in SGF can be explained mainly by the release of fluorescent beads from MPs with partially sealed pores. At the same time, the observed plateau during incubation in SGF prior to a fast release in SIF indicates the maintenance of the intact MP structure with closed pores in the simulated gastric environment. The secondary rapid change in the release profile upon exposure to SIF after 2 h of incubation in SGF can be associated with the release of fluorescent beads from (i) opening of the pH-responsive macropores or (ii) dissolution of MP polymer matrix. This step-wise release profile in response to GI tract pH conditions was similarly observed for pored MPs fabricated using o/w emulsion technique [29]. Furthermore, these studies proved the efficiency of this new fabrication protocol for developing the pored microencapsulation system.

**Fig. 4:**
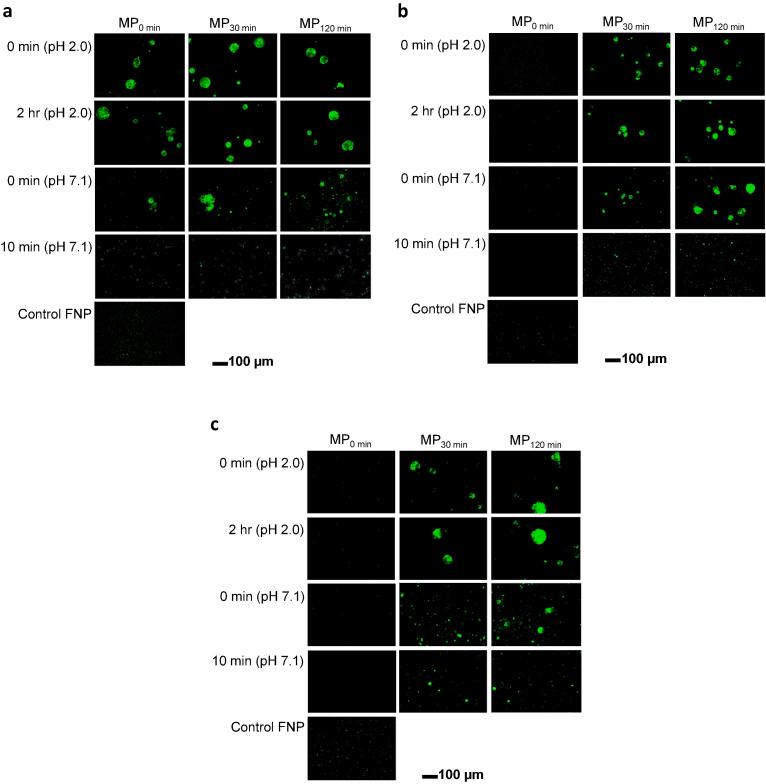
Fluorescence microscopy of fluorescent beads-encapsulated MPs. Representative fluorescence micrographs of (a) 100 nm fluorescent nanobead-, (b) 1 µm microbead-, and (c) 4 µm microbead-encapsulated MPs subjected to simulated GI tract environment (SGF: 2-h incubation at pH 2.0 and 37 °C, and SIF: 4-h incubation at pH 7.1 and 37 °C). As a control, fluorescent micrographs of fluorescent microbeads without MPs are shown for comparison.

Fluorescence microscopy analysis was performed to monitor pH-dependent release behavior of MPs over the course of incubation in the simulated GI digestive conditions. As shown in Fig. 4a, fluorescent nanobead-encapsulated MPs upheld their intact spherical morphology during the 2 h of incubation in SGF. However, when the MPs were subjected to SIF, the majority of encapsulated fluorescent beads were released from the MPs within 10 min (see Fig. 4a), which is in agreement with our observation in Fig. 3. Also, in the case of 1 µm and 4 µm beads-encapsulated MPs, majority of fluorescent beads leaked out of MPs after 10 min of incubation in SIF, as can be clearly seen by comparing the fluorescent microscope images at different time intervals to the control group (fluorescent beads only) (Fig. 4b and c). An important aspect of this observation is that the release of ingredients is not dependent on their sizes, as opposed to previous observation for emulsion-based S100 MPs [29]. Similar to the results presented in Fig. 3, this can be explained by the rapid pore opening/dissolution behavior in the neutral pH environment. Therefore, this study qualitatively illustrates that this pored MP system can encapsulate a variety of drugs (e.g., from tiny molecules to micron-sized ingredients), and shield them from gastric fluids, and then rapidly discharge them in the intestinal environment.

#### 3.2.2 β-galactosidase-encapsulated MPs

**Fig. 5:**
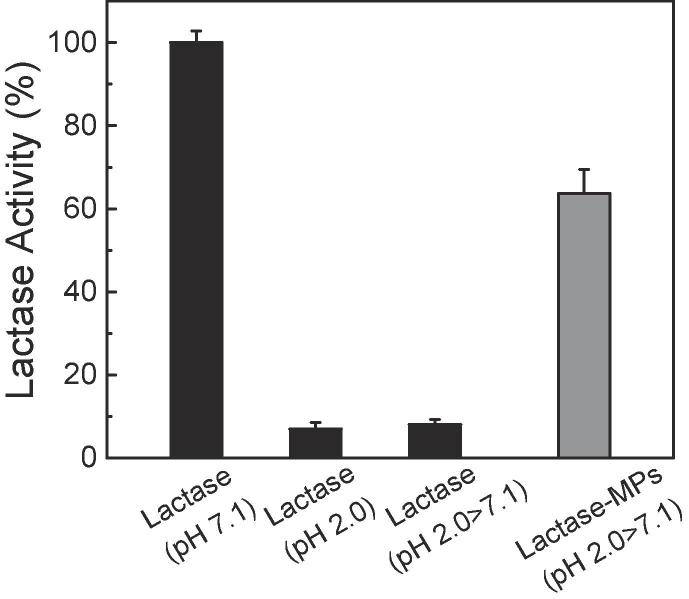
Protective effect of the MPs on lactase functional activity. The remaining activities of lactase after 4-h incubation at pH 7.1 (Lactase (pH 7.1)), lactase after 2-h incubation at pH 2.0 (Lactase (pH 2.0)), lactase after incubation in simulated GI conditions (i.e. 2-h incubation in SGF, followed by 4-h incubation in SIF; Lactase (pH 2.0 > 7.1)), and MP-encapsulated lactase after incubation in simulated GI conditions (Lactase-MPs (pH 2.0 > 7.1)). (*n* = 5, mean ± SD).

The encapsulation of fluorescent beads provided helpful information about their loading capacities, their persistent behavior in SGF environment, and their release performance in SIF environment. The results, however, do not provide information about the preservation efficiency of the MPs encapsulating biopharmaceuticals. To this end, pH-vulnerable enzyme-- β-galactosidase from Aspergillus Oryzae, was encapsulated into MP_120 min_, and its remaining activity was measured using ONPG assay in the simulated GI digestive conditions. Fig. 5 represents the activity of the released enzyme in comparison with the intact enzyme. It is noted that unprotected enzyme lost almost all its activity in the simulated gastric environment (Lactase (pH 2.0) in Fig. 5; *t*-test, *P* < 0.001) and does not recover when transferred to the simulated intestinal medium (Lactase (pH 2.0 > 7.1) in Fig. 5; *t*-test, *P* < 0.001). On the other hand, the enzyme released from the MP_120 min_ (Lactase-MPs (pH 2.0 > 7.1)) exhibited about 63.7 ± 5.8% of the activity of the intact enzyme with the same concentration, incubated only in intestinal conditions (i.e. positive control, Lactase (pH 7.1) in Fig. 5). This notable improvement in the protection efficiency of the MPs can be attributed to the maintenance of pore-closure state at acidic pH, consistent with the previous data from fluorescent beads in Fig. 3, Fig. 4. Therefore, our results further indicate that i) pore-closure efficiency by the freeze-drying process and ii) maintenance of closed pores in the gastric environment are critical factors in preserving a higher level of stability and therapeutic efficacy of MP-based drug formulations.

#### 3.2.3 Pravastatin-encapsulated MPs

The applicability of pored MPs to oral drug delivery has been further evaluated using pravastatin-encapsulated MPs, subsequent to the demonstration of the encapsulation/release behavior for fluorescent microbeads and MPs’ ability to preserve the bioactivity of the encapsulated lactase enzyme in the GI digestive conditions. Pravastatin sodium is a small molecule drug (MW: 446.51 g/mol), used to treat hypercholesterolemia [42], [43], [44]. It is water-soluble and reported to be unstable in the gastric environment. Hence, pravastatin is another excellent candidate for investigating the preservation efficiency of the MPs in GI tract conditions.

To investigate time-dependent pH response, the pravastatin release profile in the simulated GI tract environment was acquired using HPLC-MS and HPLC- UV/Vis methods [45]. As shown in Fig. S5, pravastatin sodium in SIF (pH 7.1) exhibited a signal at the retention time of 2.8–3.1 min. However, when exposed to SGF (pH 2.0), the intensity of the peak at 2.8–3.1 min decreased substantially with a generation of a new peak at the retention time of 3.3–3.7 min. The new peak corresponds to 3′α-pravastatin, the isomer of pravastatin sodium, which is associated with its degradation [41]. Thus, a correlation between the peak intensity at 2.8–3.1 min and the degree of pravastatin degradation can be used to quantitatively measure the stability of pravastatin without the interference of its 3′α isoform.

To calculate the concentration of pravastatin in the released sample, pravastatin sodium with two different concentration range in pH 7.1 buffer were tested: (1) 10–50 μg/mL with the step size of 10 μg/mL and (2) 2–10 μg/mL with the step size of 2 μg/mL (see Fig. S6a and b, respectively, for calibration curve of each condition). The pravastatin sodium concentration and HPLC-UV peak integral showed a good linear relationship.

**Fig. 6:**
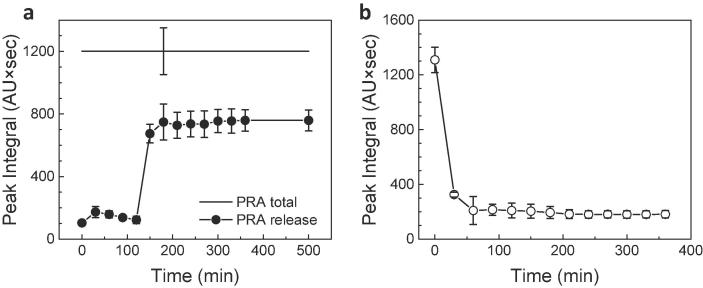
Protective effect of the MPs on pravastatin sodium stability. (a) Protection and release of the pravastatin sodium encapsulated MP formulation in simulated GI tract conditions. PRA total represents the HPLC-UV peak integral of pravastatin sodium released from MPs after incubation for 6 h at pH 7.1, followed by vortexing for 20 min to ensure complete release. (b) HPLC-UV peak integral of pravastatin sodium incubated in simulated GI tract conditions without MPs. (*n* = 5, mean ± SD).

As can be seen in Fig. 6a, pravastatin-encapsulated MPs exhibited a biphasic release profile in response to pH over time, which is same as in the fluorescent beads-encapsulated MPs. Examination of the plot reveals that 2-h incubation in SGF exhibited 8.3–16.6% of a primary leakage of pravastatin sodium (2.14–4.28 μg/mL), which can be mainly explained by the release of pravastatin sodium encapsulated into the MPs with partially sealed pores. Compared to the acidic phase, exposing MPs with pH 7.1 caused a substantial level of pravastatin release, i.e. ∼55% of the total encapsulated pravastatin, compared to the acidic phase. Then the pravastatin release reached a maximum concentration of around 61% at 1-h of incubation in SIF. After a total incubation period of 6 h, the sample was vortexed continuously for 20 min to ensure complete release of the drug from the MPs. However, no significant difference was observed compared to 1 h sample, indicating that MPs were completely dissolved within 1-h of exposure to SIF. This result shows that around 61% of the pravastatin was preserved after being incubated in a GI tract environment, which is close to 64% of the remaining activity measured from lactase-encapsulated MPs.

To verify the protective effect of MPs with macropores against acidic environmental conditions, a time-dependent degradation behavior was monitored by exposing the same concentration of pravastatin as the one encapsulated into MPs to SGF over the course of 6-h incubation. As shown in Fig. 6b, when exposed to SGF, pravastatin exhibited an abrupt decrease in its stability, followed by a gradual further decrease down to the minimum value at 1 h. As the plot shows, incubation for 30 min and 2 h in SGF resulted in the degradation of 74% and 84% of the intact pravastatin, respectively. As a result, pravastatin could maintain only 16% of remaining stability without MP formulation, which is a significantly lower level of stability than in pravastatin-encapsulated MPs.

**Fig. 7:**
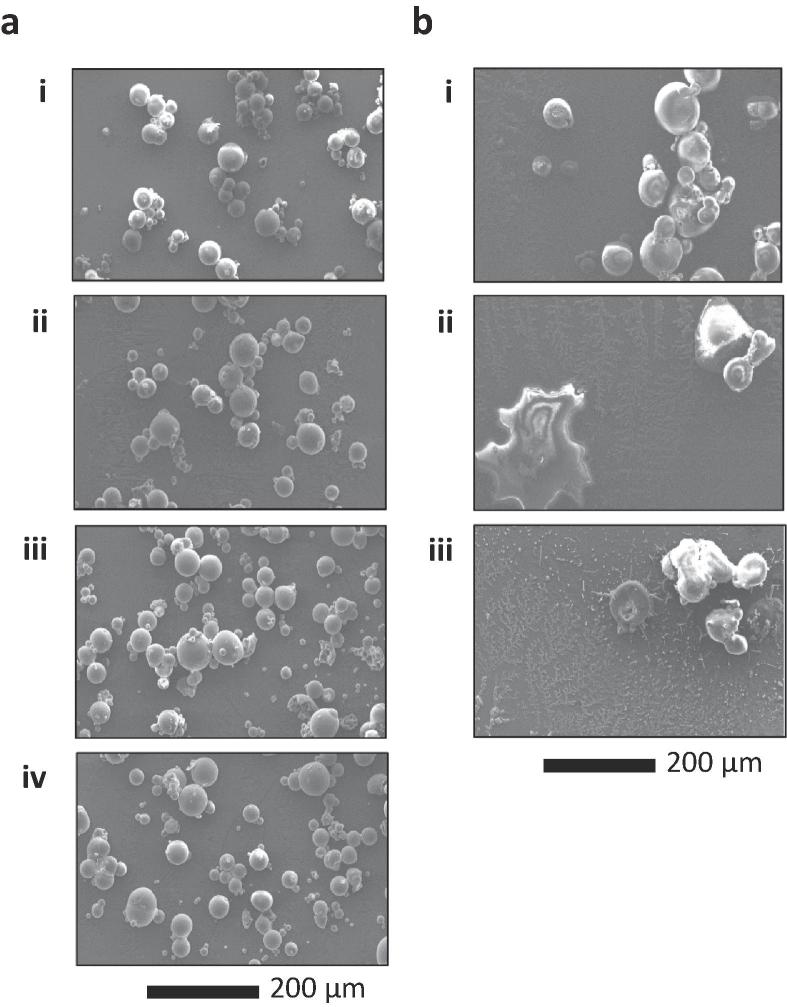
SEM images of pravastatin sodium encapsulated MPs in simulated GI tract conditions. Morphological evolution of MPs over the course of incubation time in (a) SGF (i: 0 min, ii: 30 min, iii: 60 min, and iv: 120 min), followed by (b) SIF (i: 0 min, ii: 30 min, and iii: 60 min).

SEM analysis was performed to investigate the time- and pH-dependent change in the morphology of the pravastatin-encapsulated MPs. As can be observed in Fig. 7a, MPs in SGF maintained their spherical morphology for the entire 2 h of incubation. However, MPs started to lose their structural integrity after immediate exposure to SIF, leading to near-complete dissolution as can be seen from SEM images of 30 min-/60 min-incubated samples (see Fig. 7b). This further confirms that the release of encapsulated pravastatin is associated with the morphological evolution of MPs in response to SIF.

Comparison of protective efficacy between with and without MP formulation support the conclusion that these pored MPs we developed provide a superior protection to pravastatin against harsh GI tract pH conditions (i.e. 16.0% without MP formulation vs. 61% with MP formulation). These results prove the potential of MPs with micron-sized pores and their easy fabrication method for developing environment-sensitive drug/vaccine formulations.

## 4 Discussion

An emulsion-free microencapsulation system was designed to overcome the technical challenges of the current MP-based drug delivery systems, including the exposure of drugs to organic solvents during the particle fabrication stage, loss of bioactivity in the GI tract, and low production yield. The novelty of this approach was developing a facile protocol for fabricating large-size pored MPs and using their surface pore of MPs for the encapsulation and release of drug/vaccine due to pH-dependent pore closing/opening behavior. Microemulsion method was previously applied to make a few micron-sized MPs with hundreds of nanometer-sized pores for their application in the delivery of small-molecule of drugs, having size of a virus. However, it was difficult to employ the same process to make tens of micron-sized MPs with a few micrometer-sized pores that would have the capability of encapsulating and delivering a wide range of macromolecular drugs.

To solve aforementioned technical problems, L100 MPs that have pH-responsive pores were produced by a newly modified solvent evaporation method, based on the observation that the L100 polymer powder consists of MPs with surface pores. The initial research efforts were focused to evaluate the solvent evaporation conditions for maximum pore formation and to verify the pore closure by the freeze-drying process. Under the test conditions, it was found that a longer incubation in the solvent and evaporation at a higher temperature resulted in the most promising pored MPs (i.e. MP_120 min_). The increase in the size of the surface pores can be attributed to the diffusion of a higher quantity of the organic phase into the particles during the incubation period, whose subsequent evaporation generated strong vapor flux to expand the pores [40]. Specifically, the solvent evaporation rate and degree of solvent swelling of polymers are critical factors in forming new pores and/or widening existing pores. This was confirmed through image analysis of MPs by measuring the pore/particle size ratio and the pored particles/total particles number ratio.

The controllable stimuli-sensitive surface pores can be used for direct solvent-free encapsulation of ingredients as well as their fast release at the target [29]. The extent of pore closure is critical to ensure the efficacy of drugs against the harsh acidic environment of the stomach. As shown in Fig. 1c and c, freeze-drying process induced an effective closure of a few micron-sized pores on MP surface. It is important to observe that unlike the previous findings from the emulsion-based MPs, these MPs did not exhibit ingredient size-dependent release behavior. Faster release by the L100 MPs in comparison with MPs made of S100 can be attributed to their different release mechanisms. Compared with S100 with a pKa of 6.8, L100 with a lower pKa (∼5.5) will induce instantaneous deprotonation of carboxylic groups, leading to a rapid disentanglement/dissolution of MPs in the intestinal pH. In the case of S100 MPs, ingredient release is triggered by the opening of the surface pores due to a slow dissolution of MP matrix. However, the release mechanism of L100 MPs, having easily ionizable moieties, may depend on both the dissolution of MP matrix and the pore opening. This hypothesis is supported by comparing different release patterns from S100 and L100 MPs. In the case of S100 MPs prepared by emulsion, a rapid pore opening upon exposure to SIF resulted in the release of ingredients. This explains why release rate depended on the size of ingredients (i.e. slower release for larger ingredients), as proven in the previous report [29]. On the other hand, L100 MPs with pH-responsive pores formed by solvent evaporation exhibited the burst-release mechanism. MP matrix dissolved rapidly in addition to the pore opening in SIF. Interestingly, regardless of the size of the ingredients loaded, L100 MPs displayed similar release patterns, i.e. in a size-independent manner, supporting our hypothesis (see Fig. 3). The lower pKa of the polymer will also ensure the release of the drug in the intestine since some animal studies have shown that intact S100 MPs may leave the body without releasing the drug [46], [47]. Considering that the drug release depends between the competitive mechanisms of matrix dissolution and pore opening, our observation implies that the swelling/dissolution behavior of anionic polymers through the deprotonation of the carboxylic acid groups exerts a significant impact on the release behavior of ingredients-encapsulated MPs with macropores.

The practicality of the pored MPs was evaluated by quantifying the release behavior and the remaining activity of lactase and pravastatin in the simulated GI environment. The reasons for selecting these drugs were: (1) they are completely denatured once placed in the gastric conditions (pH ≤ 2.0), (2) once denatured in the acidic conditions, they do not recover their intact structure when transferred to a neutral environment, (3) they maintain the highest level of activities in the intestinal environmental conditions. In our test conditions, about one milligram of lactase was encapsulated into one gram of MPs. The loading efficiency of the pored MP system will substantially be a function of the drug solution concentration and the amount of the added MPs. Importantly, since the encapsulation step is merely a physical process and does not induce any concern about the drug denaturation/instability, the non-encapsulated drug solution can be recovered by centrifugation process and reused by new MP powders, minimizing the loss of drugs. Therefore, it is believed that not only small drugs such as lactase and pravastatin but also many large molecule drugs that have complicated structures (e.g., vaccines) can also be encapsulated into our MPs.

All these analyses confirm the efficiency of our fabrication protocol for fulfilling the requirements of an innovative pored microencapsulation system: open pore for direct encapsulation, followed by an efficient pore closure state to preserve the loaded ingredients, and finally provide a complete release at the target sites. According to ICH guideline, dichloromethane used in MP fabrication is classified into the second group (class 2) with 6.0 mg/day of permissible daily exposure limit (PDE; concentration limit: 600 ppm) [48]. Despite the need of the systematic investigation on the residual solvent and its toxicity effects, in this work, FTIR analysis showed no evidence of residual solvent for the MP_120 min_ by comparison with spectra of MP_Original_ and DCM (Fig. S7). This supports the possibility of eliminating concern over residual organic solvent by controlling oven incubation time and temperature for MP fabrication, and freeze-drying recipe. In future, the efficiency of solvent evaporation process in pore formation and the polydispersity of the MPs fabricated using this protocol will be investigated for more favorable intestinal absorption. Also, *in vivo* demonstration will be carried to prove the efficacy of our MP system.

## 5 Conclusion

This research strives to develop a universally applicable and highly efficient oral drug delivery system, targeting small intestine. Here we report a new fabrication protocol for pored MPs that have surface pores wide enough for easily encapsulating ingredients as large as 4 µm. Also, our MP system would rapidly respond to the desired environmental pH and completely release the encapsulated drugs. The presence of the surface pore allows for the direct encapsulation of drugs and distinctively separates the MPs′ fabrication process from the drug loading step. This, in turn, resolves concerns regarding the denaturation of the drug due to its direct contact with harsh organic solvents or because of high shear stresses during emulsification process in microemulsion delivery systems. The observation of a higher level of remaining activity (>60%) of encapsulated drugs demonstrates the applicability of our pored MPs. Therefore, the system has the potential to be further investigated for the encapsulation/delivery of biomolecules and other large size ingredients.
