# Supplementary material for: Facile fabrication of microparticles with pH-responsive macropores for small intestine targeted drug formulation
Source: Eur J Pharm Biopharm. 2018 Jul;128:316–26. doi: 10.1016/j.ejpb.2018.05.014 (PMC5998383; doi:10.1016/j.ejpb.2018.05.014)
Supplement: Supplementary data 2 [file mmc2.pdf]

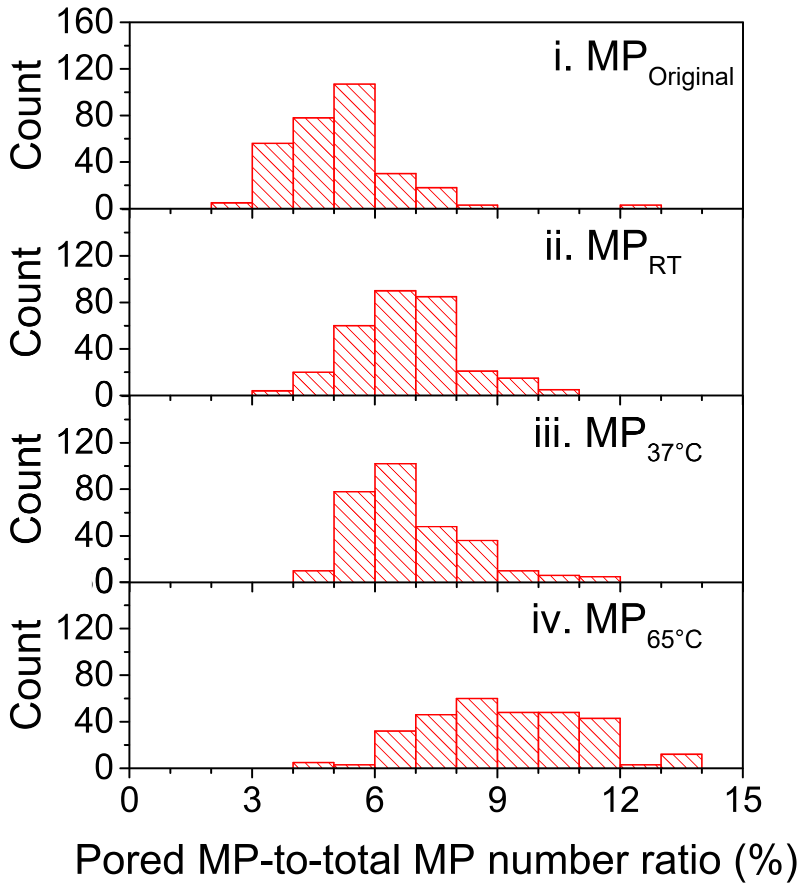


**Fig. S1** Histogram of pored MP-to-total MP number ratio of i) MP$_{Original}$, ii) MP$_{RT}$, iii) MP$_{37\ °C}$, and iv) MP$_{65\ °C}$ ($n$ = 300). The number ratio was calculated by counting the number of pored MPs and total number of MPs from each SEM micrograph. It should be noted that only MPs with pores facing upward and MPs with clearly defined pored are counted in this work.

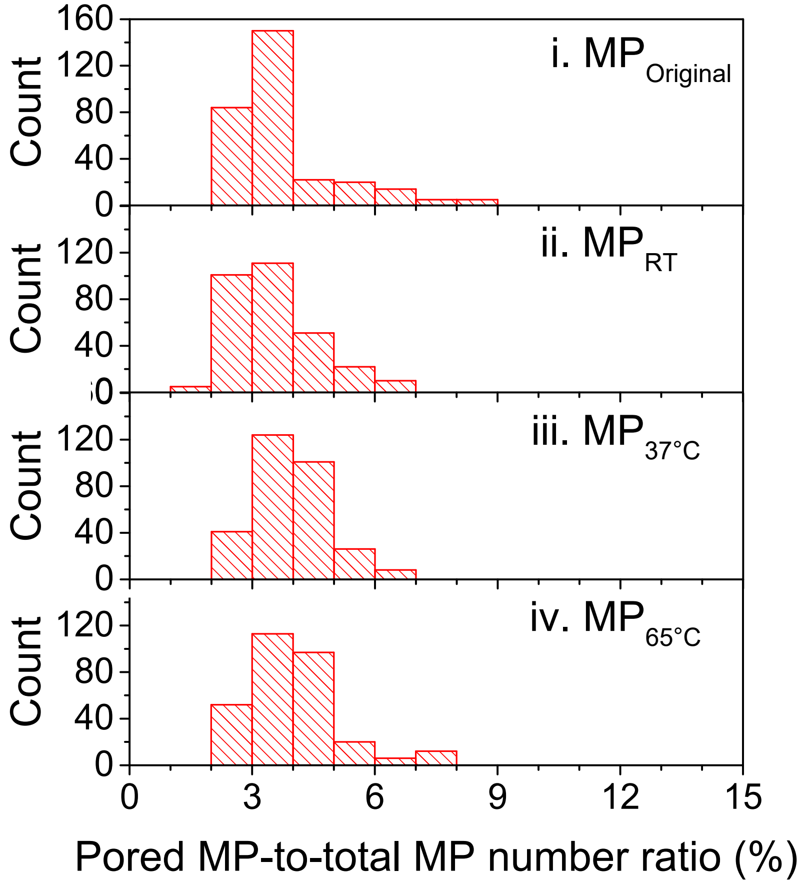


**Fig. S2** Histogram of pored MP-to-total MP number ratio of i) $MP_{Original}$, ii) $MP_{RT}$, iii) $MP_{37\ °C}$, and iv) $MP_{65\ °C}$ after freeze-drying (*n* = 300). Image analysis was performed following the same way as described in Fig. S1.

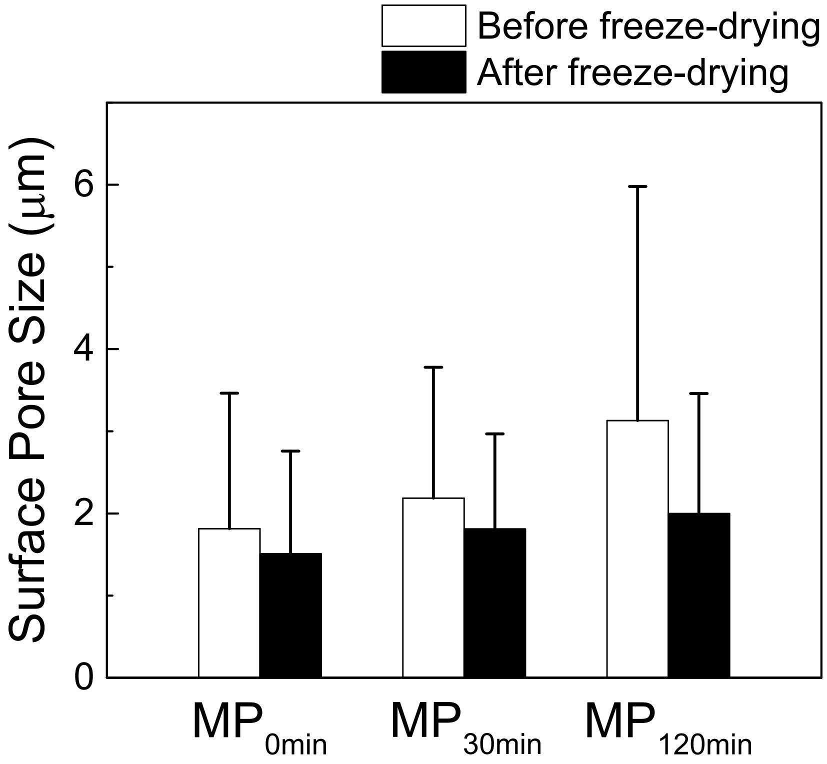


**Fig. S3** Effect of freeze-drying on the average surface pore sizes of $MP_{0\ min}$, $MP_{30\ min}$, and $MP_{120\ min}$. Since freeze-drying induces closure of pores, the pore sizes after freeze-drying in the plot represent those measured from MPs with unsealed pores. ($n$ = 300 for Before freeze-drying, $n$ = 164 for After freeze-drying, mean ± SD)

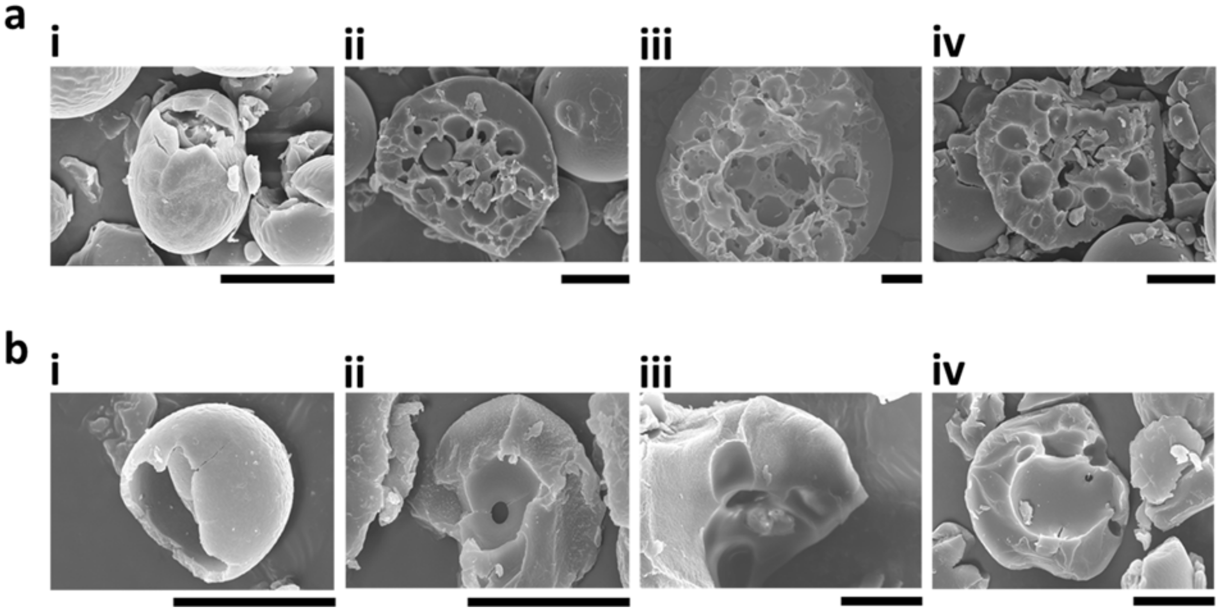


**Fig. S4** SEM images of cross-sectional view of a(i-iv) $MP_{Original}$ and b(i-iv) $MP_{120min}$. Scale bar in a and b represents 20 µm and 10 µm, respectively.

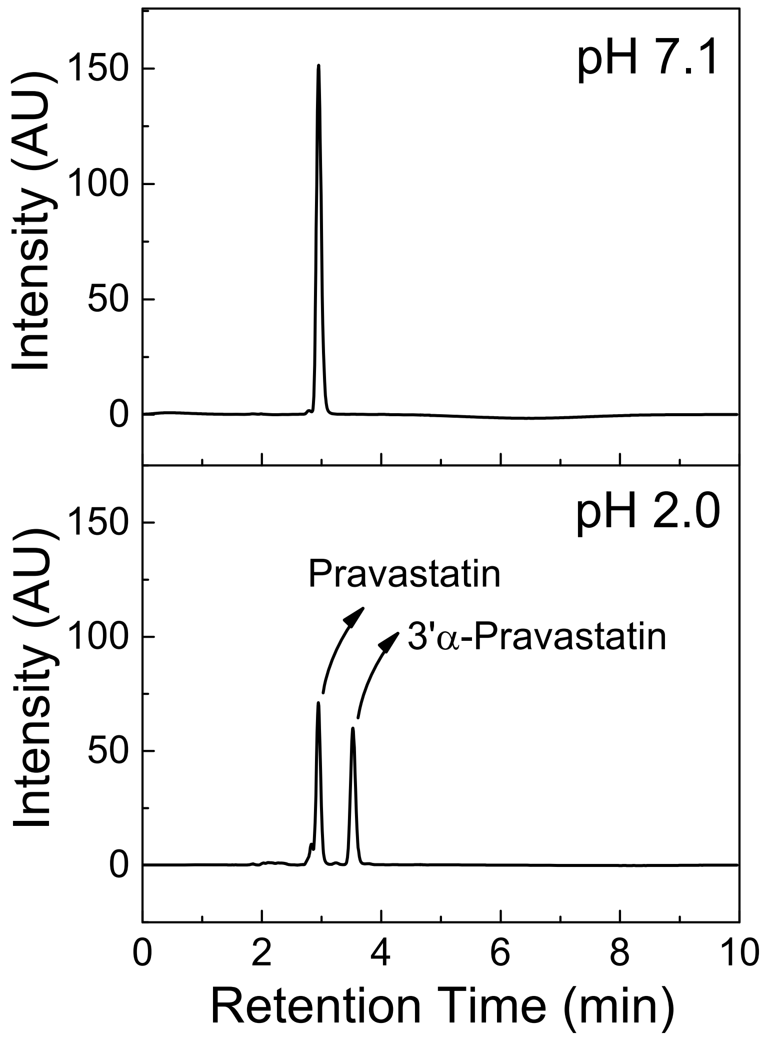


**Fig. S5** HPLC-UV spectrum of 20 μg/mL pravastatin sodium at 37°C and pH 7.1 (top), and at 37°C and pH 2.0 for 15 min (bottom).

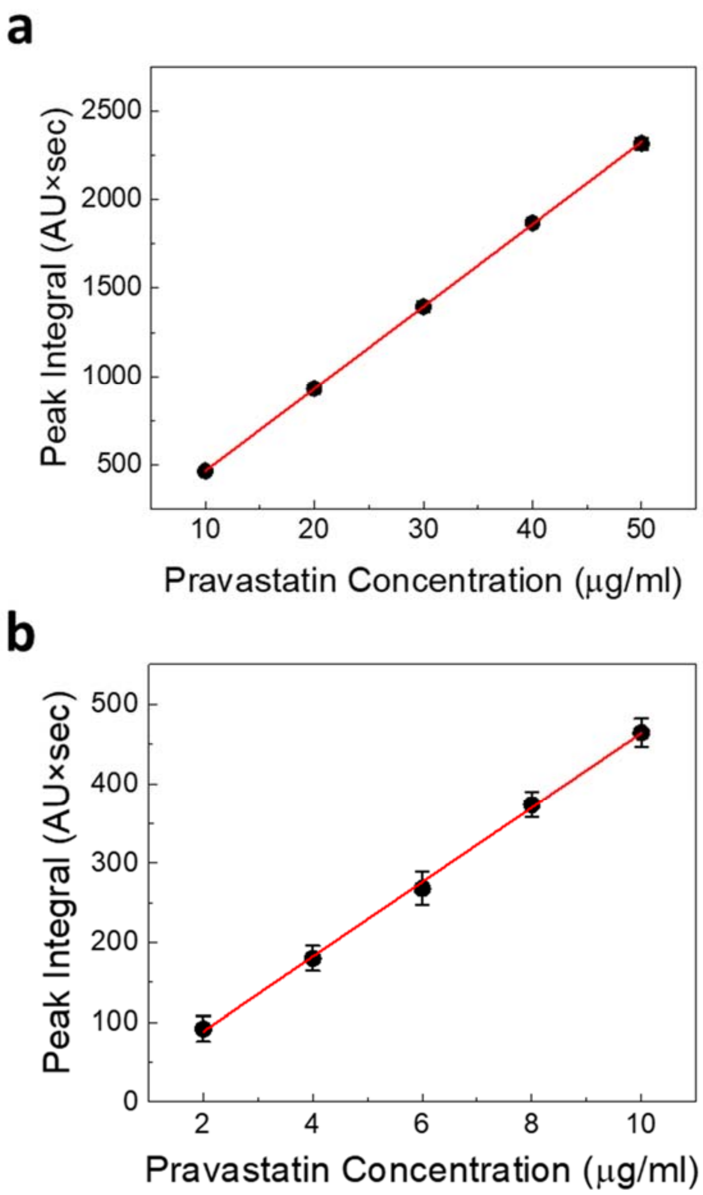


**Fig. S6** HPLC-UV calibration curve of pravastatin sodium (a) at pH 7.1 with concentration ranging from 10-50 μg/mL and step size of 10 μg/mL, and (b) at pH 7.1 with concentration ranging from 2-10 μg/mL and step size of 2 μg/mL. (*n* = 3, mean ± SD)

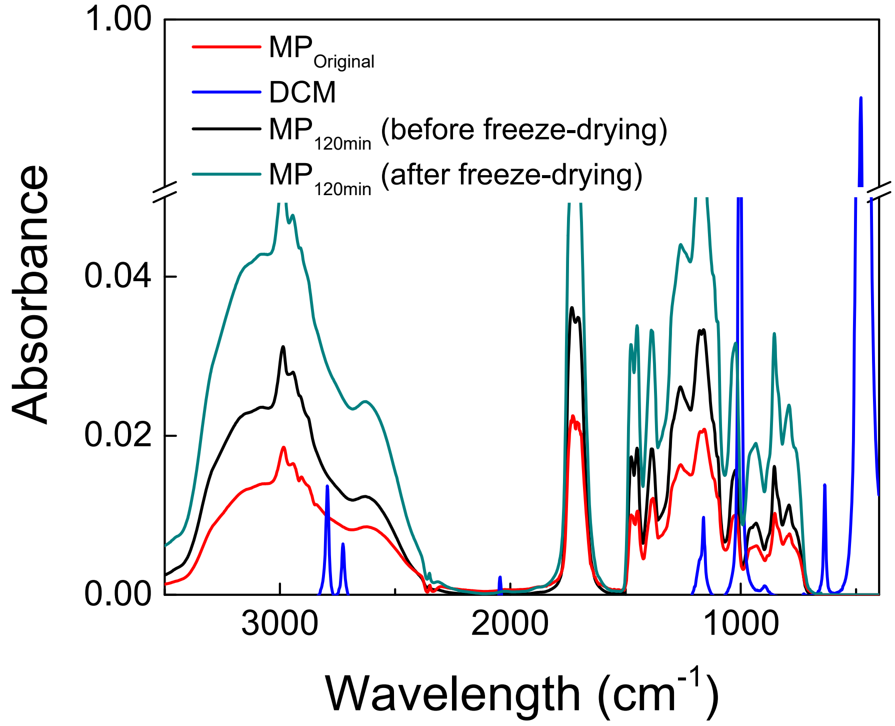


**Fig. S7** FTIR spectra of $MP_{Original}$, DCM, $MP_{120min}$ (before freeze-drying), and $MP_{120min}$ (after freeze-drying).
